# Supplementary material for: Simulation of the critical nitrogen dilution curve in Jiangxi double-cropped rice region based on leaf dry matter weight
Source: PLoS One. 2021 Nov 3;16(11):e0259204. doi: 10.1371/journal.pone.0259204 (PMC8565756; doi:10.1371/journal.pone.0259204)
Supplement: Attachment — Submitted filename: rebuttal letter.doc [file pone.0259204.s002.doc]

Dear Editors and Reviewers,

Thank you for your letter and for the reviewer’s comments concerning our manuscript entitled " Simulation of the critical nitrogen dilution curve in double-cropped rice based on leaf dry matter weight" (ID: PONE-D-21-10238). Those comments are all valuable and helpful for revising and improving our paper, as well as important guiding significance to our research. We have studied comments carefully and have made correction which we hope meet with approval. Revised portion are marked in red in the paper and the reviewers’ comments are responded as follows:

**Responses to editor:**

Editor remarks:

1. We encourage authors to add more rice varieties for this research, or repeat the research in another year or place before submitting again.

**Reply**: Thanks for the editor’s comments and suggestions.

Your comment is very good, which is worthy of our thinking and improvement. In response to your comments, Here's what we want to explain:

(1) The purpose of this paper is to study the monitoring of nitrogen concentration dilution curve of typical rice varieties in Jiangxi Province, in order to provide theoretical guidance for precise fertilization of rice in Jiangxi Province. We definitely limited the research area to double-cropping rice area in Jiangxi Province, which is clearly described in the title of the paper.
(2) In our study, the selection of rice varieties is mainly based on below: they are the local varieties or as the main push varieties from the local seed industry, take early rice Zhongjiazao 17 as an example, its 2014-2017 rice planting area in China ranked second, and which could as a representative in local rice varieties(http://www.szhgh.com/Article/gnzs/farmer/2018-11-23/185476.html).The growth period of early rice and late rice was also considered in the selection of varieties. In Experiment 1, the whole growth period of early rice: Zhongjiazao 17 was 109.0 days, and that of Changliangyou 173 was 112.0 days, that of late rice: Fumeizhan was 125.3 days, that of Taiyouhang 1573 was 121.0 days. In Experiment 2, the whole growth period of Zaoxian 618 was about 108 days, Ganxin 203 was 114.4 days, Taiyou 398 was 111.2 days, and Wufengyou T025 was 112.3 days. We carried out three field experiments in Jiangxi Province for two years and designed early and late rice experiments in each experiment, the data obtained are representative.
(3) Considering that the present study has been carried out for a long time in Jiangxi Province (and the geographical environment in the central region is similar) and the data obtained are representative. We will further expand the research of other double cropping rice areas and other varieties to see if they follow the same rules or if there are differences, the research we plan to do in our follow-up program is also in line with the good advice that experts have given us.

**Reviewer #1**

1. The biggest defect of this manuscript is that the author only used two early rice varieties and two late rice varieties to carry out the experiment. Is the sample representative enough? Is it reasonable to use the data obtained from different nitrogen gradients of two varieties for statistical analysis and model establishment.

**Reply:**

Thanks for the reviewer’s comment and suggestion.

Your comment is very good, which is worthy of our thinking and improvement. In response to your comments, Here's what we want to explain:

(1) In our study, the selection of rice varieties is mainly based on below: they are the local varieties or as the main push varieties from the local seed industry, take early rice Zhongjiazao 17 as an example, its 2014-2017 rice planting area in China ranked second, and which could as a representative in local rice varieties(http://www.szhgh.com/Article/gnzs/farmer/2018-11-23/185476.html).
(2) The growth period of early rice and late rice was also considered in the selection of varieties. In Experiment 1, the whole growth period of early rice: Zhongjiazao 17 was 109.0 days, and that of Changliangyou 173 was 112.0 days, that of late rice: Fumeizhan was 125.3 days, that of Taiyouhang 1573 was 121.0 days. In Experiment 2, the whole growth period of Zaoxian 618 was about 108 days, Ganxin 203 was 114.4 days, Taiyou 398 was 111.2 days, and Wufengyou T025 was 112.3 days.
(3) We carried out three field experiments in Jiangxi Province for two years and designed early and late rice experiments in each experiment, the data obtained are representative.
2. According to the above problems, the scope of the topics in the manuscript is too large and needs to be adjusted.

**Reply**:

Thanks for the editor’s comments and suggestions.Yes, We completely agree with your comment. It is very helpful to improve the quality of the paper. We have revised the title and relevant contents of the paper to the Jiangxi Rice region. We wonder if it is appropriate? And we would appreciate your input.

3. The ultimate goal of using nitrogen fertilizer is to improve yield. This paper analyzes the relationship between leaf dry matter weight and leaf nitrogen content, and the relationship between NNI and yield, so as to evaluate the best nitrogen application rate for high yield. However, due to the significant difference of nutrient transport efficiency among different varieties, it is suggested that the relationship between leaf dry matter weight, total N and yield should be analyzed.

**Reply**:

Thanks for the editor’s comments and suggestions.

According to your comments and suggestions, we mainly modify the article from the following two aspects:

(1) We replaced the index of leaf nitrogen concentration with that of rice plant nitrogen concentration, as shown in page10,Table 2,which are shown in the red font section of the revision.

(2)We reconstructed the power function model between leaf dry matter and rice plant nitrogen concentration, and corrected the test data in the corresponding part of model test.

The corresponding improvement part is marked in red font in the revised manuscript. (Page 10-13)

4. It is shown in Figure 4 that there are great differences in NNI among different varieties or in different growth stages of the same variety. How does the author get the conclusion that "nitrogen absorption in the double cropped rice mainly occurs in the vegetable growth stage (joining stage and booting stage), and the nitrogen nutrition status at this stage directly determines the final yield and composition"? It needs to be supported by reasonable data or references.

**Reply**:

Thanks for the editor’s comment and suggestion.

We agree with you that the NNI index has indeed changed a lot. Combined with your comment, this part has been elaborated and compiled again, and the specific revision content is as follows：

(1) We replaced the modeling parameters (plant nitrogen concentration), the result showed that the NNI index increased with the increase of n application rate, and the NNI index of the same variety varied greatly with different growth periods and different years. While from the rice jointing stage to the booting stage, the NNI index all showed an increasing trend and then decreased to the heading stage.

(2) We have improved the expression in this section to make it more concise and logical. Meanwhile, the main results of this section are added. For example, "NNI of N150 and N75 treatment were higher than or close to 1, NNI of N225 treatment was slightly higher than 1, which indicated that N150 and N75 treatment could meet the requirement of nitrogen for early rice growth".

(3) The corresponding improvement part is marked in red font in the revised manuscript. (Page 13)

5. Improve the quality of the text, such as "nitrogen nutrition index" and "NNI" in many parts of the text, which is suggested to be unified; in Table 1, the name of variety is wrongly written, such as "changiangyou 173", …… etc.

**Reply**:

Thanks for the reviewer’s comments and careful checks.

We apologize for our carelessness. We have carefully combed through the revised contents of the full text, spelling errors have been corrected line by line. The missing reference has been added to the revised manuscript.

Please see the red font in the corresponding modified position in the article.

6. The definition of the picture in the paper is poor, so it is suggested to replace the picture with higher definition.

**Reply**:

Thanks to the experts'valuable suggestions, we apologize for our carelessness.We have made corrections in accordance with the Plos One image request.

**Reviewer #2**

1. Review on the publication by Chun et al. under the title 'Simulation of the critical nitrogen dilution curve in double-cropped rice based on leaf dry matter weight.'

The article is connected with agronomically important species. The article is perfectly prepared. It's contain a lot of new and interesting results for scientific community. I do recommend it for publication

**Reply**:

Thank you for your comments and recommendations on the paper, we look forward to receiving more professional comments or suggestions from you.

We tried our best to improve the manuscript. We appreciate for Editors/Reviewers’ warm work earnestly, and hope that the correction will meet with approval. Once again, thank you very much for your comments and suggestions.

Yours sincerely,

Chun Ye; Ying Liu; Jizhong Liu; Yanda Li; Binfeng Sun, Shifu Shu

Corresponding author:Jizhong Liu

E-mail: jizhongl@163.com(L JZ)

2021.07.19
